# Heparan sulfate proteoglycan glypican-1 and PECAM-1 cooperate in shear-induced endothelial nitric oxide production

**DOI:** 10.1038/s41598-021-90941-w

**Published:** 2021-05-31

**Authors:** Anne Marie W. Bartosch, Rick Mathews, Marwa M. Mahmoud, Limary M. Cancel, Zahin S. Haq, John M. Tarbell

**Affiliations:** 1 Department of Biomedical Engineering, The City College of New York, 160 Convent Ave, New York, NY 10031 USA; 2 Department of Pathology and Cell Biology, Columbia University, New York, NY USA; 3 Taub Institute for Research on Alzheimer’s Disease and the Aging Brain, Columbia University, New York, NY USA; 4 The Knight Cardiovascular Institute, Oregon Health and Science University, Portland, OR USA

**Keywords:** Cell signalling, Glycobiology

## Abstract

This study aimed to clarify the role of glypican-1 and PECAM-1 in shear-induced nitric oxide production in endothelial cells. Atomic force microscopy pulling was used to apply force to glypican-1 and PECAM-1 on the surface of human umbilical vein endothelial cells and nitric oxide was measured using a fluorescent reporter dye. Glypican-1 pulling for 30 min stimulated nitric oxide production while PECAM-1 pulling did not. However, PECAM-1 downstream activation was necessary for the glypican-1 force-induced response. Glypican-1 knockout mice exhibited impaired flow-induced phosphorylation of eNOS without changes to PECAM-1 expression. A cooperation mechanism for the mechanotransduction of fluid shear stress to nitric oxide production was elucidated in which glypican-1 senses flow and phosphorylates PECAM-1 leading to endothelial nitric oxide synthase phosphorylation and nitric oxide production.

## Introduction

Endothelial cells (ECs) coat the inner surface of blood vessels and regulate vascular function in response to blood flow-induced fluid shear stress. Production of the local vasodilator, nitric oxide (NO), by ECs in response to fluid shear stress is of particular importance in the maintenance of vascular health. NO production proceeds via the mediating enzyme endothelial nitric oxide synthase (eNOS), which converts l-arginine and molecular oxygen to NO in the presence of several cofactors, including tetrabiopterin (BH_4_). This action is increased in response to elevated physiologic fluid shear stress, which activates eNOS by phosphorylation^1^. NO is anti-atherogenic, suppressing the development of plaque and clot formation in blood vessels^2–4^. Great efforts have been made in recent years to understand how ECs regulate flow-induced NO, beginning with the flow sensors at the blood-cell surface interface^5,6^.

Kuchan et al. showed that the shear-induced NO response of endothelial cells has an early phase (seconds to minutes after a step increase in shear) that is calcium and G-protein dependent as well as a later phase (minutes to hours) that is independent of calcium and G-protein^7,8^. There has been considerable emphasis on the early phase mechanotransduction mechanisms and the Ca^2+^ channels that are activated by shear, including the PIEZO1 channel^9^ and the TRPV4 channel^10^. In addition, de la Paz et al., in early phase experiments, showed that there is a PECAM-1-Gαq/11 mechanosensitive complex that contains an endogenous heparan sulfate proteoglycan that is critical for regulating the flow response^11^. The present work emphasizes the later phase mechanism that is most relevant to the sustained production of NO over longer times.

The transmembrane protein platelet endothelial cell adhesion molecule 1 (PECAM-1) is abundant at the intercellular junctions between ECs and is often used as a marker of EC phenotype. It is also associated with shear dependent NO production as studies have shown loss of flow sensitive production of NO when PECAM-1 is knocked down^12,13^. The loss of PECAM-1 in bovine aortic ECs (BAECs) and human umbilical vein ECs (HUVECs) led to reduced phosphorylation of eNOS (ser1177) and NO production following 30 min of flow^12,13^. These studies indicate that PECAM-1 is a regulator of shear-induced NO. But whether PECAM-1 senses flow directly to initiate NO production or responds to a primary sensor of shear stress has not yet been determined.

PECAM-1 has been studied previously as a mechanosensor, attuned to the small forces shear stress imparts on the protein, and shown to initiate cellular signaling pathways in response to the application of force^14–16^. When anti-PECAM-1 functionalized magnetic beads were used to apply force to PECAM-1 on the surface of porcine aortic ECs for 30 min, Akt (protein kinase B) became activated^14^. Akt is an upstream regulator of NO synthase, cyclooxygenase-2 (COX-2) production, and cell viability and is downstream of phosphatidylinositol 3-kinase (PI3K)^17,18^. Based on these findings that PECAM-1 is responsive to force application and may regulate signaling pathways intersecting with those guiding vasodilator release, the present study was in part designed to investigate whether force applied to PECAM-1 induces EC production of NO. Atomic force microscopy (AFM) pulling with anti-PECAM-1 functionalized probes was used to apply force for 30 min to ECs. NO production was detected using the DAF-2 DA fluorescent dye. The DAF-2 DA indicator was also used to confirm prior findings that PECAM-1 knockdown in human ECs blocks shear-induced NO production^12,13^.

The role of endothelial glycocalyx components, specifically surface protein glypican-1 and its bound glycosaminoglycan (GAG) heparan sulfate (HS), have been investigated recently in shear-induced NO and prostacyclin (PGI_2_) production^19,20^. AFM pulling on confluent rat ECs with anti-glypican-1 or anti-HS functionalized probes elicited increased NO production^19^. Additionally, glypican-1 siRNA transfection or HS enzymatic removal in bovine, rat, and human cells blocks shear-induced NO production^21–23^ but has no effect on shear-induced PGI_2_ or COX-2 production^20,22^. Based on these insights, the present study also asked whether AFM pulling with anti-glypican-1 functionalized probes initiates NO production in human ECs and whether that force-sensitivity is reliant on the presence of PECAM-1.

PECAM-1 is known to interact with other mechanisms to sense changes in shear stress^11,16,24^. It has also been observed that HS and glypican-1 accumulate at cell–cell junctions, where PECAM-1 is localized after shear stress application^25^. A HS-to-PECAM-1 binding complex has been observed^26,27^ that is involved in endothelial mechanotransduction in the early phase (1 min exposure to shear)^11^. Human coronary artery ECs (HCAECs) with HS enzymatically degraded or reconstructed with PECAM-1 mutants missing HS binding domains had inhibited (early phase) shear-induced Akt phosphorylation—a precursor to NO production^11^.

PECAM-1 forms binding complexes through extracellular Ig-like domains, but also participates in signaling by activation of its intracellular tail that has two tyrosines, 686 and 663, that may be phosphorylated by mechanical force, including shear stress^28,29^. PECAM-1 phosphorylation is activated by kinases of the Src family, notably c-Src^12,28,29^. Blocking PECAM-1 phosphorylation with PP1, a Src kinase inhibitor, blocks eNOS phosphorylation in response to shear^12^, demonstrating that PECAM-1 phosphorylation is essential for shear-induced NO production through eNOS phosphorylation. The main objective of the present study is to determine how PECAM-1 phosphorylation is triggered by fluid shear stress and in the process identify the upstream shear sensor for sustained NO production. The overall goal of this study therefore is to elucidate the mechanotransduction pathways for shear-induced NO production, which will improve our understanding of cardiovascular disease progression and enable the design of future therapeutics to rescue endothelial dysfunction.

## Results

### AFM pulling on glypican-1 but not PECAM-1 induces NO production in HUVEC

To test that NO production can be induced using AFM pulling in HUVEC, the same methods as previously described^19^ were repeated in human endothelial cells. First, adhesion forces were measured using silicon nitride AFM cantilevers with pyramidal tips to confirm probe-HUVEC interaction. Probes functionalized with anti-glypican-1 and anti-PECAM-1 had adhesion forces of 130 pN and 165 pN, respectively, significantly higher than the adhesion force of the rabbit IgG isotype control (Supplemental Figure S1).

**Figure 1:**
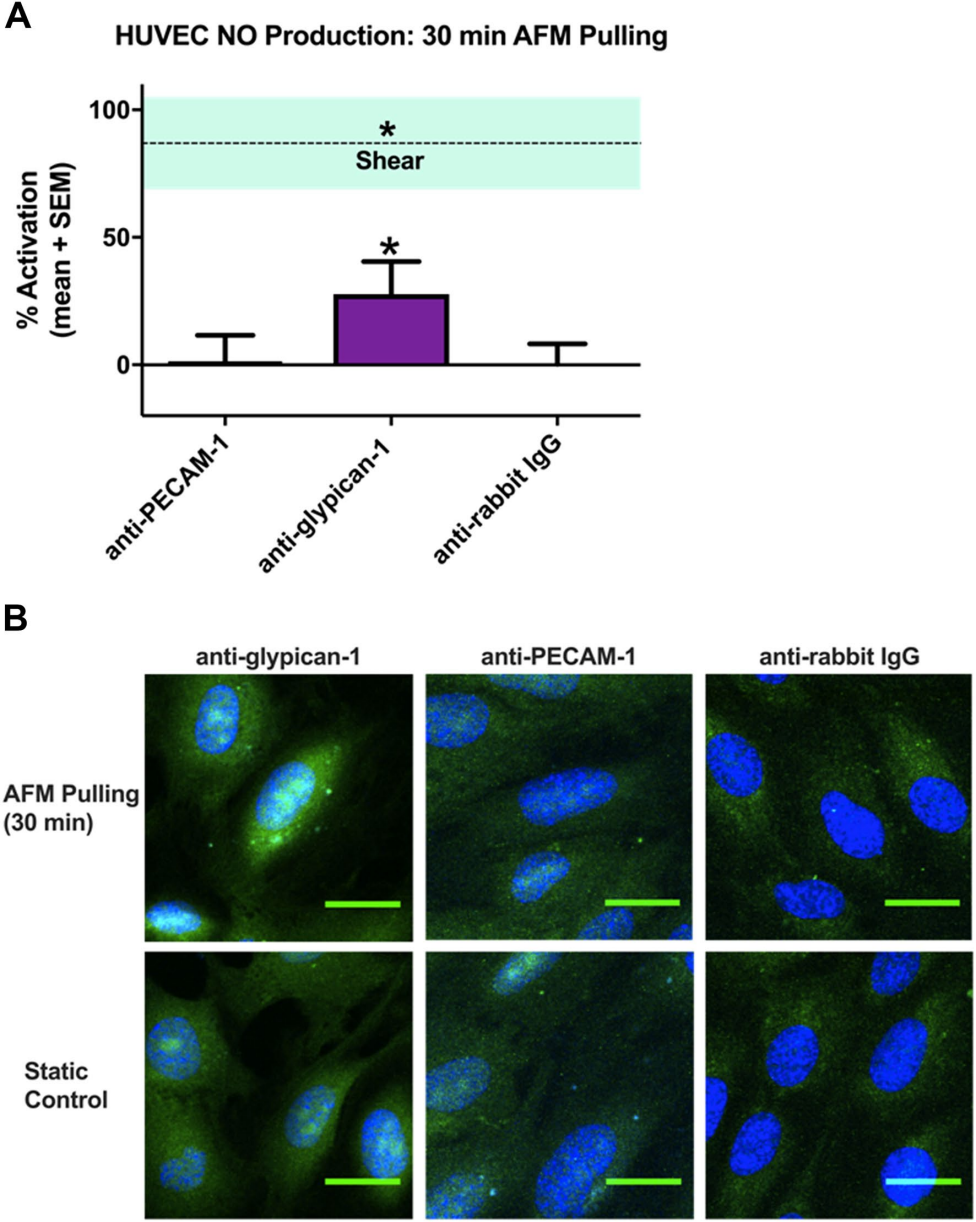
AFM pulling on glypican-1, but not PECAM-1, induced NO production in HUVEC. HUVEC monolayers were probed with rabbit anti-glypican-1, rabbit anti-PECAM-1, or normal rabbit IgG tipless AFM cantilevers. (**A**) Mean % activation ± SEM are shown (n = 27 for all groups). *P < 0.05 versus static conditions (0% activation) using two-tailed one-sample t-test. The dotted line is mean % activation of NO ± SEM for cells stimulated with fluid shear stress for 30 min (n = 16). (**B**) Representative confocal max intensity Z stack projections are shown for HUVEC with DAF 2T (green) fluorescence counterstained with DAPI (blue). The top row shows cells probed with functionalized AFM tips, while the bottom row shows unprobed cells from the same slide, approximately 2 mm away from probing location.

Confluent HUVEC were stimulated with rabbit anti-glypican-1, rabbit anti-PECAM-1, and anti-rabbit IgG isotype control functionalized tipless AFM probes for 30 min (Fig. 1). The 30 min time point was chosen because a significant reduction in NO production after 30 min of shear stress with PECAM-1 depletion has been observed in HUVEC^12^. Pulling on the HUVEC surface for 30 min with glypican-1 probes induced 28% mean activation, significantly increased from static control regions located 2 mm from the probing site on the same slide. The same method using PECAM-1 probes did not yield significant activation; neither did pulling with the isotype control. Free glypican-1 antibody was also tested for its ability to stimulate NO production without the application of a pulling force. 30 min incubation with anti-glypican-1 had no significant effect on HUVEC NO production compared to untreated controls (Supplemental Figure S3).

To confirm the HUVEC results, NO production after pulling with PECAM-1 probes was measured in another cell line, rat fat pad endothelial cells (RFPEC). AFM pyramidal probes functionalized with PECAM-1 antibody exhibited strong binding to the RFPEC surface (Supplemental Figure S2). However, tipless triangular cantilevers functionalized with PECAM-1 antibody did not induce activation of NO production (Supplemental Figure S4). ‘Pointed’ probes (triangular cantilevers with pyramidal tips) were also functionalized with the same PECAM-1 antibody to test whether a smaller tip shape would yield activation, but pulling with pointed PECAM-1 probes had similarly insignificant activation. Anti-goat-IgG functionalized tipless triangular cantilevers included as isotype controls did not induce significant activation. We conclude that AFM pulling with anti-PECAM-1 probes has no significant effect on NO production (Supplemental Figure S4).

COX-2 production was also measured in RFPEC to serve as a positive control for PECAM-1 pulling, validating the efficacy of PECAM-1 pulling as a sufficient mimic of shear-induced mechanical force. Shear-induced COX-2 production was initially assessed using immunofluorescent staining in RFPEC monolayers after 30 min of fluid shear stress application (Supplemental Figure S5A) and found to be increased significantly with shear (dashed line in Supplemental Figure S5B). Pulling on RFPEC with tipless AFM probes functionalized with PECAM-1 antibody for 30 min resulted in COX-2 activation (Supplemental Figure S5B). Isotype control and bare probe pulling had no significant effect on COX-2 production. Although prior studies have shown that the glycocalyx is not active in shear-induced COX-2 production^20^, AFM pulling was also conducted using a glypican-1 antibody functionalized probe and no significant effect on COX-2 production was observed (Supplemental Figure S5B). This is consistent with prior work showing that glycocalyx-degrading enzymes do not affect shear-induced PGI_2_ or COX-2 production^20,22^. These negative findings also show that force application to the cell membrane through surface proteins that are not involved in the relevant signaling cascade does not induce COX-2 production.

### Production of NO is impaired in glypican-1 knockout mice

**Figure 2:**
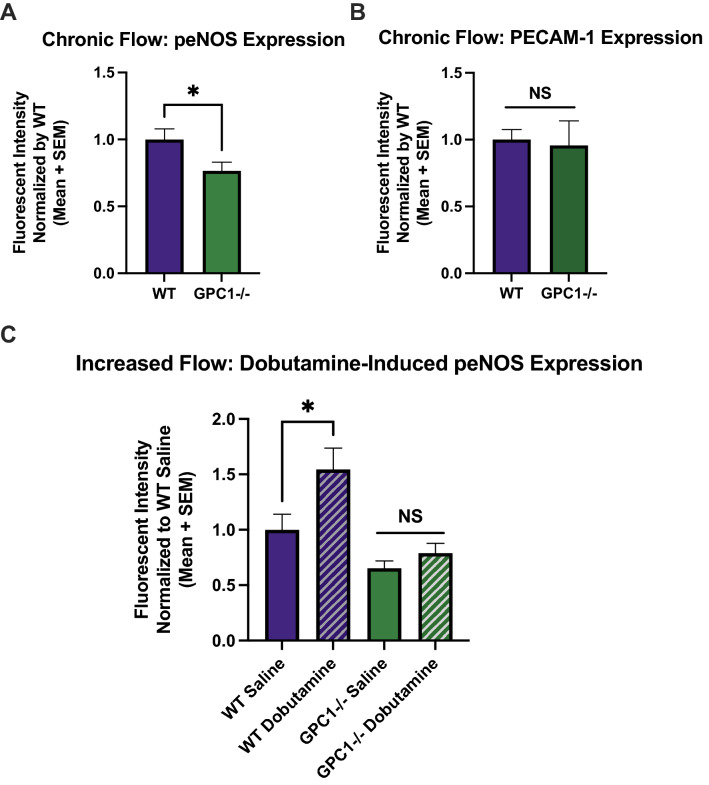
Glypican-1 knockout mice have inhibited eNOS phosphorylation in both chronic and increased flow conditions. Descending aorta from WT and Gpc1−/− mice were en face immunostained for peNOS and PECAM-1. (**A**) Mean normalized peNOS expression under chronic flow is shown for WT (n = 10) and Gpc1−/− mice (n = 10). *P < 0.05 using two-tailed student’s t-test. (**B**) Mean normalized PECAM-1 expression under chronic flow is shown for WT (n = 6) and Gpc1−/− (n = 5) mice. *P < 0.05 using two-tailed student’s t-test. (**C**) Mean normalized peNOS expression under increased flow induced by dobutamine for 30 min is shown for WT (n = 9 and 8 for saline and dobutamine groups, respectively) and Gpc1−/− mice (n = 8 for both saline and dobutamine groups). *P < 0.05 using one-way ANOVA followed by Sidak’s multiple comparisons test.

Glypican-1 knockout mice (Gpc1−/−) were bred to further characterize the relationship between glypican-1 and NO production in an animal model. Reduced glypican-1 expression was confirmed by immunostaining in Gpc1−/− mice (Supplemental Figure S6). Since phosphorylation of eNOS is required for NO production, we measured peNOS expression in Gpc1−/− mice. Immunostaining revealed that Gpc1−/− mice had significantly reduced peNOS expression in the descending aorta (Fig. 2A). Mean peNOS expression in Gpc1−/− mice normalized to mean WT expression was 0.77. PECAM-1 expression was not significantly different from that of WT control mice (Fig. 2B). In a second experiment, mice were administered dobutamine, a β-adrenergic agonist that has been shown to increase cardiac flow in mice^30^. WT animals treated with dobutamine for 30 min had significantly increased peNOS expression compared to WT animals treated with saline sham (Fig. 2C). Normalized mean peNOS expression was 1.54 in dobutamine treated WT mice. However, Gpc1−/− mice given dobutamine had no significant change in peNOS (Fig. 2C).

### PECAM1 siRNA transfection blocks shear-induced NO production in HUVEC

**Figure 3:**
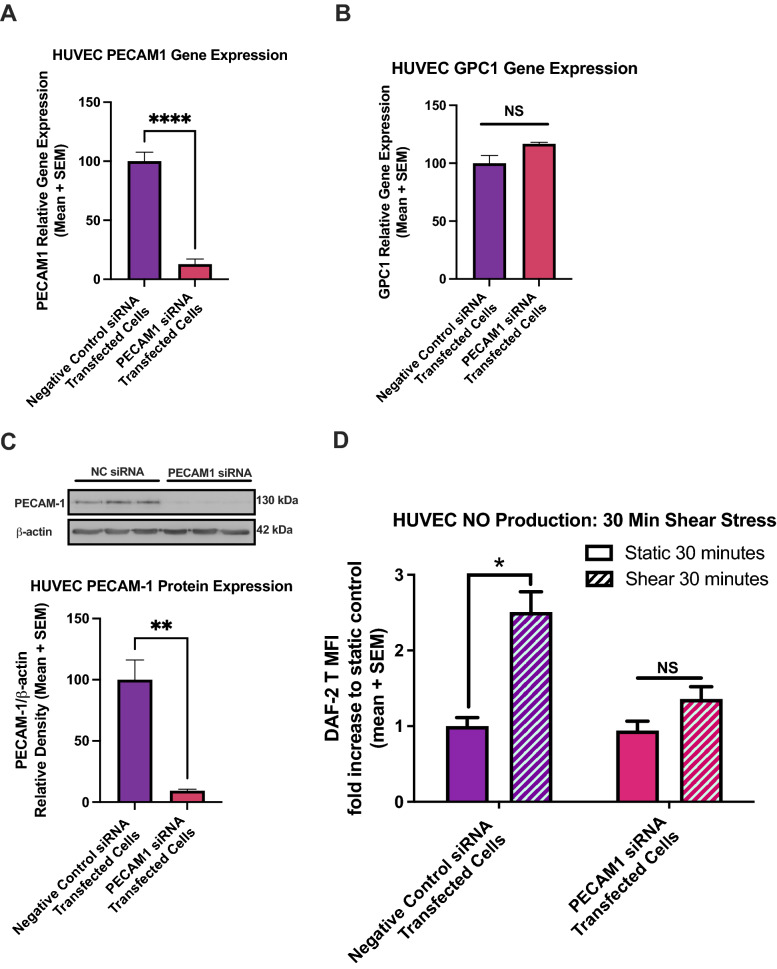
PECAM-1 knockdown blocks shear-induced NO production at 30 min in HUVEC. (**A**) PECAM1 gene expression is shown in cells transfected with negative control siRNA and with siRNA specific to PECAM1 mRNA (n = 10 for each group). Gene expression was normalized to GAPDH and β-ACTIN expression levels and both transfection groups were normalized to the negative control expression data, set to 100% expression. *P < 0.05 using two-tailed student’s t-test. (**B**) GPC1 gene expression is shown in transfected cells (n = 4 for each group). Expression levels did not significantly change using two-tailed student’s t-test. (**C**) PECAM-1 protein expression in transfected cells (n = 3 for each group) and corresponding western blot. *P < 0.05 using two-tailed student’s t-test. Full length blot is presented in Supplemental Figure S8. (**D**) DAF-2T normalized levels are shown for transfected HUVEC exposed to 30 min of fluid shear stress. From left to right, n = 8, 16, 8, 16. *P < 0.05 using one-way ANOVA followed by Sidak’s multiple comparisons test.

To confirm the role of PECAM-1 in force-induced NO production, siRNA transfections were conducted to deplete PECAM1 mRNA and protein in HUVEC, and shear stress was applied using the 6-well rotating disc apparatus. Real time-PCR (qPCR) showed a significant decrease in PECAM1 gene expression (to 2%) for HUVEC transfected with PECAM1 siRNA compared to those transfected with a duplex containing a randomized sequence not present in humans, termed “Negative Control siRNA” and set to 100% (Fig. 3A). Glypican-1 expression was not significantly affected by PECAM1 siRNA; GPC1 mRNA did not significantly change in HUVEC transfected with PECAM1 siRNA compared to controls (Fig. 3B). Western blot confirmed a lack of protein production, showing a significant decrease (to 9%) in PECAM-1 protein expression for HUVEC transfected with PECAM1 siRNA (Fig. 3C). NO was again measured using DAF-2 DA and the shear-induced DAF-2 T levels were normalized to static control levels for each set. Fluid shear stress induced a 2.51-fold increase in NO production in negative control transfected cells compared to static control (Fig. 3D). In PECAM-1 knockdown cells, there was no significant change in static NO production, with a mean value of 0.94-fold compared to negative control transfected cells. After 30 min of shear stress, PECAM-1 knockdown cells did not significantly increase their NO production, with a mean increase of only 1.36-fold, which was not significantly different from that of static PECAM-1 knockdown cells (Fig. 3D).

### PECAM1 siRNA transfection blocks NO production elicited from AFM pulling on glypican-1

**Figure 4:**
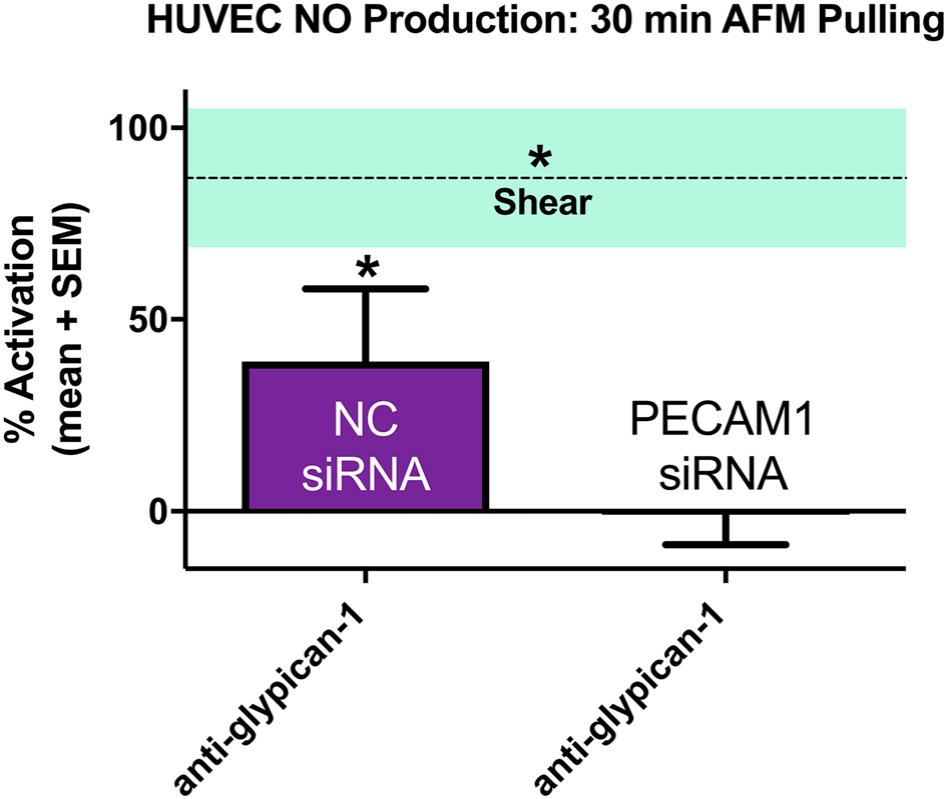
AFM pulling with glypican-1 antibody on PECAM-1 deficient HUVEC does not induce NO production. HUVEC monolayers were probed using AFM pulling with rabbit anti-glypican-1 tipless cantilevers. Mean % activation of NO production and SEM are shown for negative control siRNA transfected cells (n = 27) and PECAM1 siRNA transfected cells (n = 27). The dotted line marks the mean % activation of NO ± SEM for cells stimulated with fluid shear stress for 30 min (n = 16). *P < 0.05 versus static conditions (0% activation) using two-tailed one-sample t-test for bars and two-tailed student’s t-test for shear line.

To further understand the effect of PECAM-1 knockdown on the ability of endothelial cells to produce NO in response to mechanical force, AFM pulling on glypican-1 was conducted in HUVEC transfected with control and PECAM1 targeted siRNA (Fig. 4). Control cells stimulated with AFM pulling by anti-glypican-1 functionalized probes exhibited a significant increase in NO production, with 39% mean activation, consistent with that shown in Fig. 1 for untreated HUVEC. In contrast, PECAM1 siRNA transfected cells stimulated with AFM pulling by anti-glypican-1 functionalized probes did not have any significant response (Fig. 4).

### Blocking HS-to-PECAM-1 extracellular interaction does not affect shear-induced NO production

**Figure 5:**
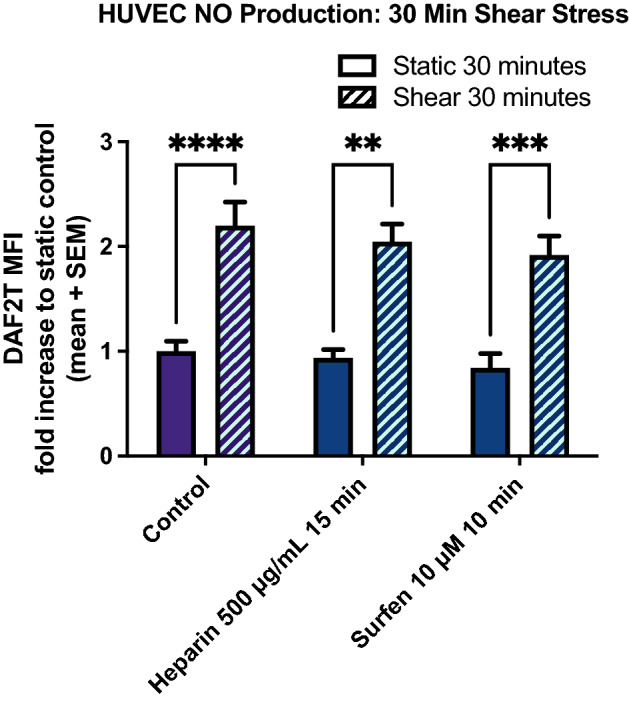
Heparin and Surfen treatment does not affect shear-induced NO production at 30 min in HUVEC. Heparin (500 µg/mL) and surfen (10 µM) were used to treat HUVEC 15 min prior to fluid shear stress exposure to block shear-induced HS binding to PECAM-1. DAF-2T levels were normalized to static control levels within each set. From left to right, n = 17, 22, 8, 15, 12, 16. *P < 0.05 using one-way ANOVA followed by Sidak’s multiple comparisons test.

The data in Figs. 1, 2, 3, 4 imply that there is an interaction between glypican-1 and PECAM-1 and that glypican-1 is upstream of PECAM-1. To understand the interaction between PECAM-1 and glypican-1 in shear-induced NO production, heparin and surfen blocking treatments were used to perturb extracellular interactions via HS chains. Figure 5 shows shear-induced NO production in HUVEC after 30 min of shear with heparin and surfen treatments (Fig. 5). In untreated HUVEC, 30 min of shear caused a 2.20-fold change in NO production. When heparin was used to treat HUVEC monolayers for 15 min prior to shear induction, static and shear NO levels were 0.94-fold and 2.05-fold, respectively. Similarly, when surfen was used to treat HUVEC monolayers for 10 min prior to shear induction, static and shear NO levels were 0.68-fold and 1.58-fold, respectively. Neither heparin nor surfen had any effect on the shear-induced increase in NO production (Fig. 5). To confirm the lack of change from heparin or surfen treatment, these experiments were repeated in RFPEC and similar trends were observed (Supplemental Figure S7).

### Cleaving HS at the HUVEC surface blocks shear-induced PECAM-1 activation via tyrosine phosphorylation

**Figure 6:**
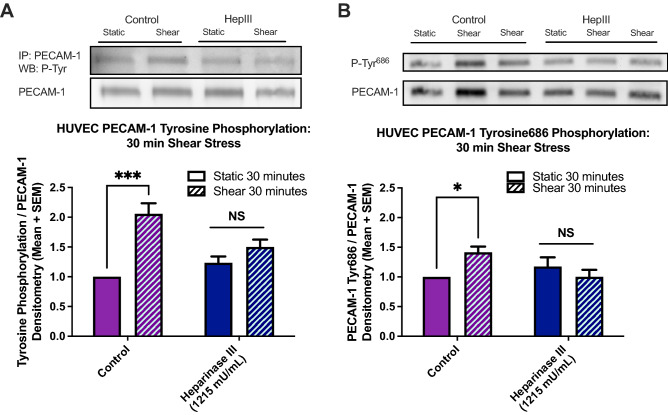
HS cleavage blocks shear-induced PECAM-1 tyrosine kinase phosphorylation at 30 min in HUVEC. Heparinase III (1215 mU/mL) was used to cleave HS from HUVEC monolayers 2 h prior to fluid shear stress exposure. (**A**) PECAM-1 phosphorylation levels were measured using western blotting for total tyrosine phosphorylation (following PECAM-1 immunoprecipitation) and (**B**) for PECAM-1 specific tyrosine 686 phosphorylation. Full length blots are presented in Supplemental Figure S8. Densitometry values from both phosphorylation types were normalized to PECAM-1 expression in each sample and then normalized to untreated static control levels within each set. From left to right within each set, (**A**) n = 6, 13, 5, 14 for total tyrosine phosphorylation (**B**) n = 6, 12, 6, 12 for tyrosine 686 phosphorylation. Results in each graph are averaged from 5 or more independent experiments. *P < 0.05 using one-way ANOVA followed by Sidak’s multiple comparisons test.

To investigate intracellular interactions between the glycocalyx and PECAM-1, heparinase III cleaving enzyme was used to remove HS (the only GAG on glypican-1) from the surface of HUVEC and total tyrosine phosphorylation of PECAM-1 was assessed (Fig. 6). Untreated HUVEC exhibited a 2.10-fold increase in shear-induced PECAM-1 activation compared to untreated static control cells (Fig. 6A). HUVEC treated with heparinase III had only a 1.50-fold increase after fluid shear stress was applied that was not significantly different from that of static heparinase treated controls.

PECAM-1 tyrosine residue 686 (Tyr686) was investigated in the same manner to understand how a specific phosphorylation site on PECAM-1 was affected by heparinase III treatment (Fig. 6B). Untreated HUVEC exhibited 1.42-fold increase in shear-induced PECAM-1 Tyr686 activation compared to untreated static control cells. HUVEC treated with heparinase III had no significant increase in levels of PECAM-1 Tyr686 activation after fluid shear stress was applied.

## Discussion

The goal of the present study was to elucidate the roles of PECAM-1 and glypican-1 in vascular flow-induced mechanotransduction of NO. Similar to prior work, fluid shear stress was applied to confluent monolayers and NO production activated globally across the monolayer surface was measured. Additionally, novel AFM pulling methods were applied to PECAM-1 and glypican-1 individually to measure NO production activated locally by individual surface protein molecules. Glypican-1’s role in NO production was further confirmed in glypican-1 knockout animals. A significant effort was focused on understanding the interdependence between PECAM-1 and glypican-1 in the production of NO by repeating AFM pulling experiments on PECAM-1 knockdown cells.

Interestingly, we discovered that PECAM-1 and glypican-1 participate cooperatively in the mechanotransduction of fluid shear stress. While the concept of cooperative mechanosensing is not new^31^, cooperation between glypican-1 and PECAM-1 has not been described previously. AFM pulling with glypican-1 probes induced a significant increase in NO production in human cells, consistent with prior work in rat cells^19^, that was blocked in PECAM-1 knockdown cells. Yet direct pulling on PECAM-1 in wild type cells did not elicit a NO response. These findings were consistent across experiments in two cell types, HUVEC and RFPEC. Additional pulling with pointed AFM probes was also conducted as a second approach to ensure access to junctional regions where PECAM-1 is localized and, similarly, did not elicit a NO response. Confirming these in vitro observations, glypican-1 knockout mice had reduced levels of phosphorylated eNOS at baseline as well as after dobutamine-induced increases in blood flow compared to WT mice.

**Figure 7:**
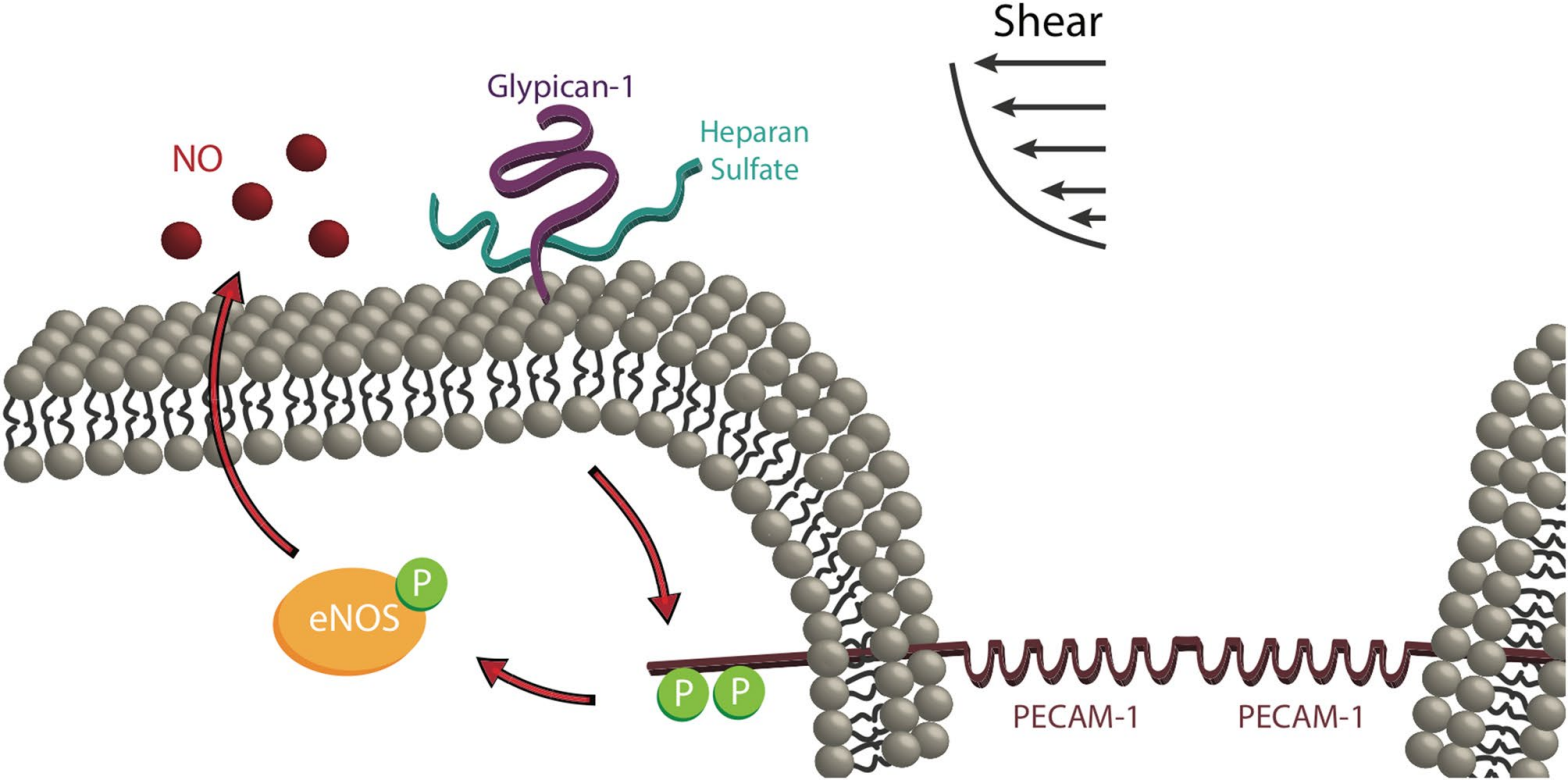
Shear-induced NO production via heparan sulfate and glypican-1 mechanotransduction and PECAM-1 phosphorylation leading to eNOS activation and NO synthesis. Fluid shear stress is initially sensed by heparan sulfate proteoglycan glypican-1. That signal is transduced to the intracellular tail of PECAM-1 protein. PECAM-1 becomes activated by tyrosine phosphorylation. PECAM-1 activation triggers phosphorylation of eNOS and NO production increases. NO diffuses out of the cell through the membrane to engage in autocrine or paracrine signaling.

Having established that glypican-1 and PECAM-1 act in series to produce NO, and glypican-1 is the upstream shear sensor, we further investigated the mechanism linking HS proteoglycan glypican-1 and PECAM-1. No effect was observed on NO production by blocking extracellular binding between HS and PECAM-1 with heparin and surfen. Shear-induced intracellular PECAM-1 activation was also assessed after HS removal from the EC luminal surface. We used heparinase III to selectively remove HS from the EC surface^32^ to block shear stress activation of glypican-1. Since HS is the only GAG on glypican-1, removal of HS blocks shear-induced glypican-1 signaling. This is consistent with earlier studies that showed heparinase III treatment blocked shear-induced NO production in EC^21^. Of course, removal of HS also blocks shear-induced syndecan-1 signaling, but we showed previously that direct pulling on syndecan-1 with AFM probes does not elicit a NO response^19^. We observed that heparinase III-treated HUVEC have diminished shear-induced PECAM-1 tyrosine phosphorylation, with specific impairment occurring at Tyr686. Based on this HS-dependent PECAM-1 activation and the PECAM-1-dependent glypican-1 mechanosensing pathway, we conclude that fluid shear stress sensed through the heparan sulfate chains of glypican-1 is transduced to the interior of the cell, initiating a biochemical signaling cascade that phosphorylates PECAM-1. Activation of PECAM-1 leads to phosphorylation of eNOS^13,31^, which ultimately increases NO production. This process is outlined in the cartoon below, summarizing our overall findings (Fig. 7).

Further work is necessary to explore the intermediate signaling molecules controlling glypican-1 to PECAM-1 activation, but examining prior studies dealing with the signaling to and from these proteins allows us to form a clearer image of how this pathway may function. It is well established that PECAM-1 is activated by c-Src^12,28,29^. Furthermore, Bianco et al*.* found that glypican-1 and c-Src colocalize in lipid rafts on mouse mammary epithelial cells and that an epidermal growth factor binds to glypican-1 inducing activation of cytoplasmic c-Src^33^. There is also significant evidence for PECAM-1-to-eNOS signaling. PECAM-1 is associated with eNOS in the cell membrane of wild type mice^31^ and is necessary for the shear-induced phosphorylation of eNOS at Ser1177^12,34^.

Shear stress likely plays a role not only in stimulating glypican-1 mechanically, but in amplifying the NO response by increasing the cooperation between glypican-1 and PECAM-1. Past work has shown that HS and glypican-1 (not syndecan-1) move toward cell junctions in response to 30 min of shear stress, a response that is mediated by glypican-1’s GPI-linkage to mobile lipid rafts, which also reorganize toward the cell junction within 30 min of shear^25^. The colocalization of eNOS and PECAM-1 has also been shown to increase with shear stress and peak at the 30 min time point investigated in this study^12^. Thus the detailed mechanism for cooperation between glypican-1 and PECAM-1 in shear-induced NO production discovered here may include glypican-1’s GPI-linkage to lipid rafts and associated intracellular eNOS movement to cellular junctions in response to fluid shear, forming a signaling complex with PECAM-1, and more rapidly engaging in intracellular signaling to activate PECAM-1’s cytoplasmic tail and engage in eNOS phosphorylation.

## Methods

### Cell culture

HUVEC (Lonza C2519AS) were statically cultured at 37 °C with 5% CO_2_ in EGM-2 (Lonza CC-3162), plated on fibronectin coated surfaces, and grown to confluency. At least 1 h prior to the onset of experiments, culture media was changed to experimental media consisting of phenol red-free, serum-free DMEM supplemented with 1% bovine serum albumin (BSA, Sigma A7284). For fluorescent monitoring of NO production, 5 µM DAF-2 DA (Sigma D225) and 1 µM NO cofactor tetrahydrobiopterin (BH4, Sigma T4425) were added to experimental media and incubated for 40 min prior to stimulation.

To investigate whether extracellular binding between PECAM-1 and HS influence shear-induced NO release, heparin and surfen blocking treatments were employed. When added to cell culture media, heparin competitively binds to the extracellular Ig-like domains of PECAM-1 because of its structural similarity to HS and reduces HS-to-PECAM-1 binding^35–37^. Surfen (bis-2-methyl-4-amino-quinolyl-6-carbamide) is a small molecule antagonist of HS that disrupts HS binding to other proteins^12,31,33,38^. Heparin and surfen treatments were used in the present study to block HS-to-PECAM-1 extracellular binding and shear-induced NO production was measured. When blocking treatments were used they were added to experimental media: 2 h 1215 mU/mL heparinase III (Ibex 50-012-001) treatment^12,31–33,38^ was conducted prior to DAF-2 DA incubation and stimulation while 10 min 500 µg/mL heparin (Sigma H3149-10KU) and 10 µM surfen (Sigma S6951) treatments^11,39,40^ were added following DAF-2 DA incubation and prior to stimulation.

### Mice

All animal protocols were approved by the Institutional Animal Care and Use Committee (IACUC) of The City College of New York, all experiments were performed in accordance with relevant guidelines and regulations, and reporting follows the recommendations in the ARRIVE guidelines. A pair of heterozygous mutants on the CD1 background (Gpc1−) was a gift from Arthur Lander’s lab^41^. Gpc1- were bred to produce homozygous mutants (Gpc1−/−) and wild types (Gpc1+/+). To assess the effects of increases in blood flow 8-week-old mice were treated with dobutamine hydrochloride (Cat. No. 0515; Tocris), following previously published methods^20^. Briefly, mice were given a single intraperitoneal injection of Dobutamine at a dose of 1.5 μg/g body weight, vehicle control mice were given an equivalent volume of 0.9% saline at 5 μl/g body weight. Following 30 min of Dobutamine injection, mice were euthanized by CO_2_ asphyxiation and the vasculature was fixed by pressure perfusion as previously described^42^. Briefly, a mid-line surgical incision was made from the abdominal wall to the thoracic wall and the heart was exposed. The inferior vena cava and right atrium were severed and 30 mL of PBS containing 1% BSA were pressure perfused to clear the blood. The vessels were then pressure perfused for 5 min with PBS containing 2% paraformaldehyde. The aorta was dissected and stored in PBS until immunostained.

### PECAM1 siRNA transfection

Cells were plated for transfection experiments in antibiotic-free culture media and transfected 24 h later using 10 µM siRNA duplexes (IDT) via lipofectamine RNAiMax (Invitrogen 13778). Gene expression was assessed via quantitative real-time PCR (RT-qPCR) 24 h after transfection. Protein expression was assessed via western blotting 72 h after transfection. Shear stress and AFM studies were also conducted 72 h after transfection in experimental media.

### Gene expression using RT-qPCR

RNA was isolated and purified from transfected cells using RNeasy kit (Qiagen) according to the manufacturer’s protocol. Purified RNA was converted to cDNA by reverse transcription using Omniscript RT kit (Qiagen). RT-qPCR was performed using SYBR Green PCR Master Mix (Applied Biosystems) on the ABI PRISM 7000 sequence detection system (Applied Biosciences) to determine PECAM1 siRNA knockdown efficiency and check for off-target effects on glypican-1. The expression of housekeeping genes β-actin and GAPDH were used to normalize PECAM1 and GPC1 gene expression.

### Protein expression using western blotting

After shear experiments or 72 h after siRNA transfection, cells were collected for protein expression analysis. Cell lysis buffer consisted of 1× RIPA buffer (Millipore 20-188) supplemented with 1 mM sodium orthovanadate and cocktails of protease (Thermo Scientific 88666) and phosphatase (Roche 4906845001) inhibitors. Cell extracts were collected in RIPA buffer on ice and centrifuged at 10,000×*g* for 10 min at 4 °C to pellet and discard cellular debris. Lysates used in immunoprecipitation studies were first incubated with anti-PECAM-1 primary antibody (Proteintech 11265-1-AP) for 1 h at 4 °C, then Protein A/G PLUS-Agarose was added and the mixture was incubated overnight at 4 °C on a rocker platform. Immunoprecipitates were collected by centrifugation at 1000×*g* for 5 min at 4 °C and washed with PBS. Pellets were mixed with sample buffer, boiled 3 min, and loaded to 7% handcast SDS-PAGE gels. Protein content of lysates was determined using Bradford protein determination assay and 30 µg of total sample protein was mixed with sample buffer, heated to 100 °C for 5 min, and loaded to 7% hand-cast SDS-PAGE gel. Protein bands were separated by electrophoresis and transferred to PVDF membranes using standard methods. Membranes were blocked with 5% BSA in TBS-T solution at room temperature for 45 min. Primary antibodies were diluted in blocking solution and incubated at 4 °C on a rocker platform overnight. Anti-PECAM-1 (Proteintech 11265-1-AP), anti-phosphotyrosine clone 4G10 (Millipore 05-321), anti-PECAM-1 Tyr713 (Sigma SAB4503968-100UG), and β-actin (Sigma A5441) antibodies were used at 1:1,000. Tyrosine 713 is a synonym for tyrosine 686 in the mature PECAM-1 protein, once the 27 amino acid long signal peptide is cleaved. Secondary antibody was used at 1:10,000 and diluted in 3% BSA TBS-T solution.

### Fluid shear stress application

Six-well rotating flat cylindrical disc and plate systems were used to apply 20 dynes/cm^2^ fluid shear stress via steady laminar flow to cultured EC in a cell culture incubator as described previously^43^.

### AFM force mapping and tensile force application

An Asylum MFP-3D atomic force microscope (AFM) was used to conduct experiments following the same procedure as recently published^19^. Briefly, silicon nitride cantilevers with pyramidal tips (k = 0.09 N/m, Asylum Research BL-TR400PB) were functionalized with antibodies and used to determine adhesion forces across several regions of the confluent cell monolayer. Flexible PEG linker molecules (NHS-PEG-Acetal linkers from Linz, Austria) were used to tether anti-glypican-1 (Proteintech 16700-1-AP), anti-PECAM-1 (Proteintech 11265-1-AP), and anti-rabbit IgG (Cell Signaling 2729S) antibodies to probes. Silicon nitride tipless cantilevers (k = 0.08 N/m, Nanoworld PNP-TR-TL) with the same functionalization scheme and antibodies were used with DAF-2 DA loaded cell monolayers to measure induced NO. Fibronectin coated glass gridded slides (EMS 63405-01) were used to track force mapping location for subsequent fluorescence imaging and comparison to unprobed regions 2 mm away from probing site on the same slide. The AFM force map settings were as previously described^19^. In adhesion force experiments with pyramidal tips 3 nN maximum indentation force was used, while in DAF-2 induction experiments 10 nN maximum indentation force was used with 10 s dwell time at the endothelial surface. Indentation (z-axis) speed was 1.6 µm/s and XY speed was 400 nm/s in all cases. Probing area in both studies was 20 µm × 20 µm and the number of indentations per scan was adjusted to fit 30-min total elapsed time (10 points × 9 lines).

### Immunofluorescence and confocal imaging of cultured cells

Following mechanical stimulation with either fluid shear stress application or AFM force application, cell monolayers loaded with DAF-2 DA and expressing DAF-2 T were fixed in 2% paraformaldehyde (PFA) for 10 min, counterstained with DAPI, and mounted for confocal imaging. Cyclooxygenase-2 (COX-2) staining was also conducted after mechanical stimulation. COX-2 staining included 4% PFA fixation, 2 h in blocking solution (1% BSA, 10% goat serum, 0.3 M glycine, 0.1% Tween-20), overnight incubation with primary antibody (1:40 rabbit anti-cyclooxygenase-2, Abcam 15191) at 4 °C in blocking solution (1% BSA, 10% goat serum), 1 h incubation with secondary antibody (1:500 goat anti-rabbit IgG Alexa Flour 488, Invitrogen A11008) at room temperature in blocking solution (1% BSA, 10% goat serum), and DAPI counterstaining. Zeiss LSM 710 laser scanning confocal microscope was used to collect Z-series stacks. Using MATLAB (Mathworks, USA), maximum intensity Z-projections were created and the point of intersection between static and activated regions on pixel intensity histogram curves were used to threshold images before contrast stretching back to original size. Mean fluorescent intensities (MFIs) were calculated from the average grey value of each of these images and MFIs were normalized by the average MFI of static control samples. To calculate percent activation, normalized MFIs were converted to percentages by subtracting 1 and multiplying by 100.

### En face immunostaining of the mouse endothelium

The expression of specific proteins was assessed at the endothelium of the thoracic descending aorta by *en face* immunostaining. Fixed arteries were processed for immunostaining using specific antibodies for phospho-eNOS (Cat. No. 074281, Millipore), PECAM-1 (553373, BD Pharmingen), glypican-1 (Cat. No. SC66909, Santa Cruz) and the nuclei were stained using DAPI (Cat. No. D1306, Thermo Fisher Scientific). The stained tissue was mounted on a microscope slide *en face,* followed by confocal laser imaging using a ZEISS LSM 800 microscope. Protein expression was quantified as fluorescent intensity using Image J.

### Data analysis

Results are presented as mean + SEM for all data sets and differences were considered significant when p < 0.05. GraphPad Prism was used to perform statistical analyses. When comparing two groups, statistical analyses were performed using unpaired two-tailed student’s t-test. When comparing one group to an artificially homogenous group (such as activation percentages for AFM probed regions vs 0% static regions), one sample t-test was used. When comparing more than two groups, one-way ANOVA was performed followed by the Sidak method for multiple comparison testing.

## Supplementary Information

Supplementary Information.

## Data Availability

All data generated or analysed during this study are included in this published article (and its Supplementary Information files).
