# Supplementary material for: Heparan sulfate proteoglycan glypican-1 and PECAM-1 cooperate in shear-induced endothelial nitric oxide production
Source: Sci Rep. 2021 May 31;11:11386. doi: 10.1038/s41598-021-90941-w (PMC8166914; doi:10.1038/s41598-021-90941-w)

Supplementary Information

Heparan Sulfate Proteoglycan Glypican-1 and PECAM-1 Cooperate in Shear-Induced Endothelial Nitric Oxide Production

Anne Marie W. Bartosch[1,2,3], Rick Mathews[1,4], Marwa M. Mahmoud[1], Limary M. Cancel[1], Zahin S. Haq[1], John M. Tarbell[1*]

1. Department of Biomedical Engineering, The City College of New York, NY, USA
2. Department of Pathology and Cell Biology, Columbia University, New York, NY, USA
3. Taub Institute for Research on Alzheimer's Disease and the Aging Brain, Columbia University, New York, NY, USA
4. The Knight Cardiovascular Institute, Oregon Health and Science University, Portland, OR, USA

Running title:
Glypican-1 and PECAM-1 Cooperate in NO Production

Corresponding author:
John Tarbell, Ph.D.
Department of Biomedical Engineering
Steinman Hall, 275 Convent Ave
The City College of New York
New York, NY, 10031
jtarbell@ccny.cuny.edu

Corresponding author during production process:
Limary Cancel
Department of Biomedical Engineering
The City College of New York
160 Convent Ave
New York, NY, 10031
limarycancel@yahoo.com

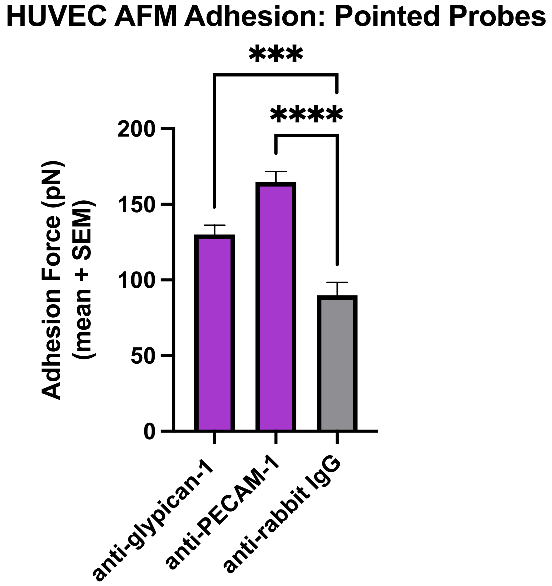

Figure S1. AFM Adhesion Force between Functionalized AFM Probes and HUVEC Monolayer. Human confluent monolayers were probed with AFM triangular cantilevers with pyramidal tips functionalized with rabbit glypican-1 (n = 63) and rabbit PECAM-1 (n = 63) antibodies and their isotype control anti-rabbit IgG (n = 53). *P<0.05 vs. isotype control using one-way ANOVA followed by Sidak's multiple comparison testing.

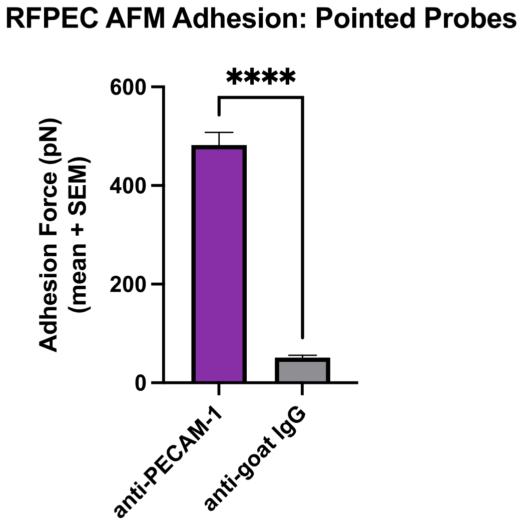

Figure S2. AFM Adhesion Force between Functionalized AFM Probes and RFPEC monolayer. RFPEC confluent monolayers were probed with AFM triangular cantilevers with pyramidal tips functionalized with goat PECAM-1 antibody (n = 57) and its isotype control anti-goat IgG (n = 128). *P<0.05 vs. isotype control using student's two-tailed two-sample t-test.

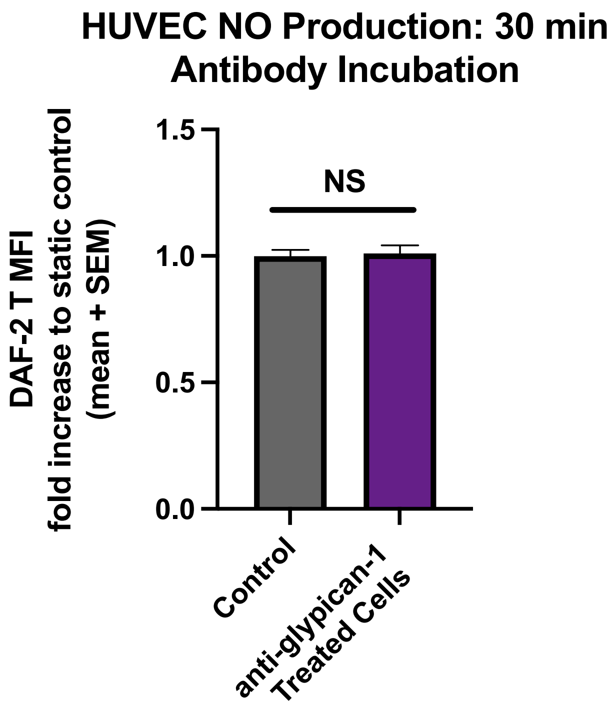

Figure S3. Glypican-1 Antibody Binding Has No Effect on Nitric Oxide Production in HUVEC. HUVEC monolayers were incubated with 1:100 anti-glypican-1 for 30 minutes. DAF-2 T levels were normalized to untreated control levels. No significant change was detected using student's two-tailed two-sample t-test (n = 25 for both groups).

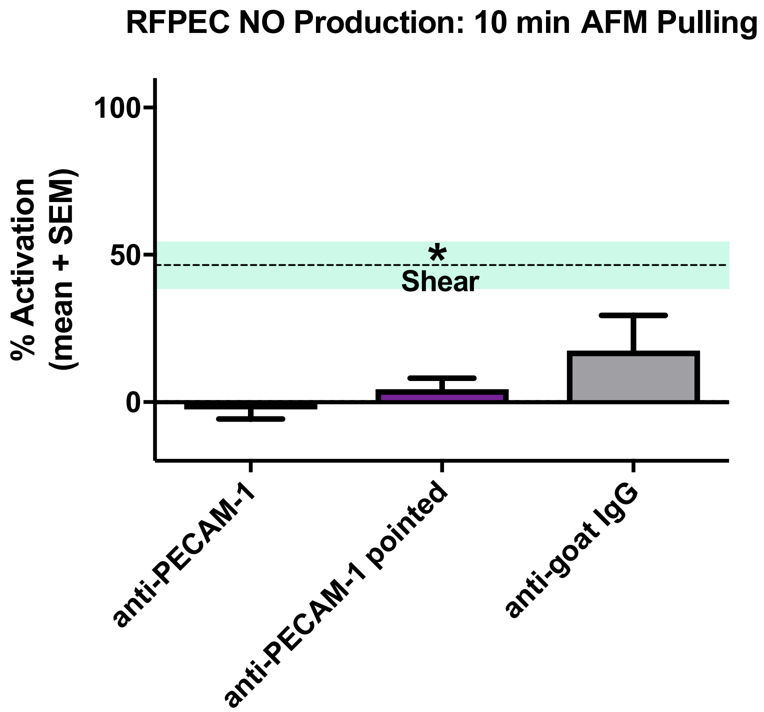

Figure S4. AFM Pulling with PECAM-1 Antibody Has No Effect on Nitric Oxide Production in RFPEC. RFPEC monolayers were probed with goat anti-PECAM-1 or normal goat IgG tipless AFM cantilevers and goat anti-PECAM-1 pyramidal AFM tips ('pointed'). Mean % activation and SEM are shown in the bar graph for n = 12, 7, 13 from left to right. *P<0.05 versus static conditions (0% activation) using two-tailed one-sample t-test. The dotted line is mean % activation of NO +/- SEM for cells stimulated with fluid shear stress for 10 minutes (n = 20).

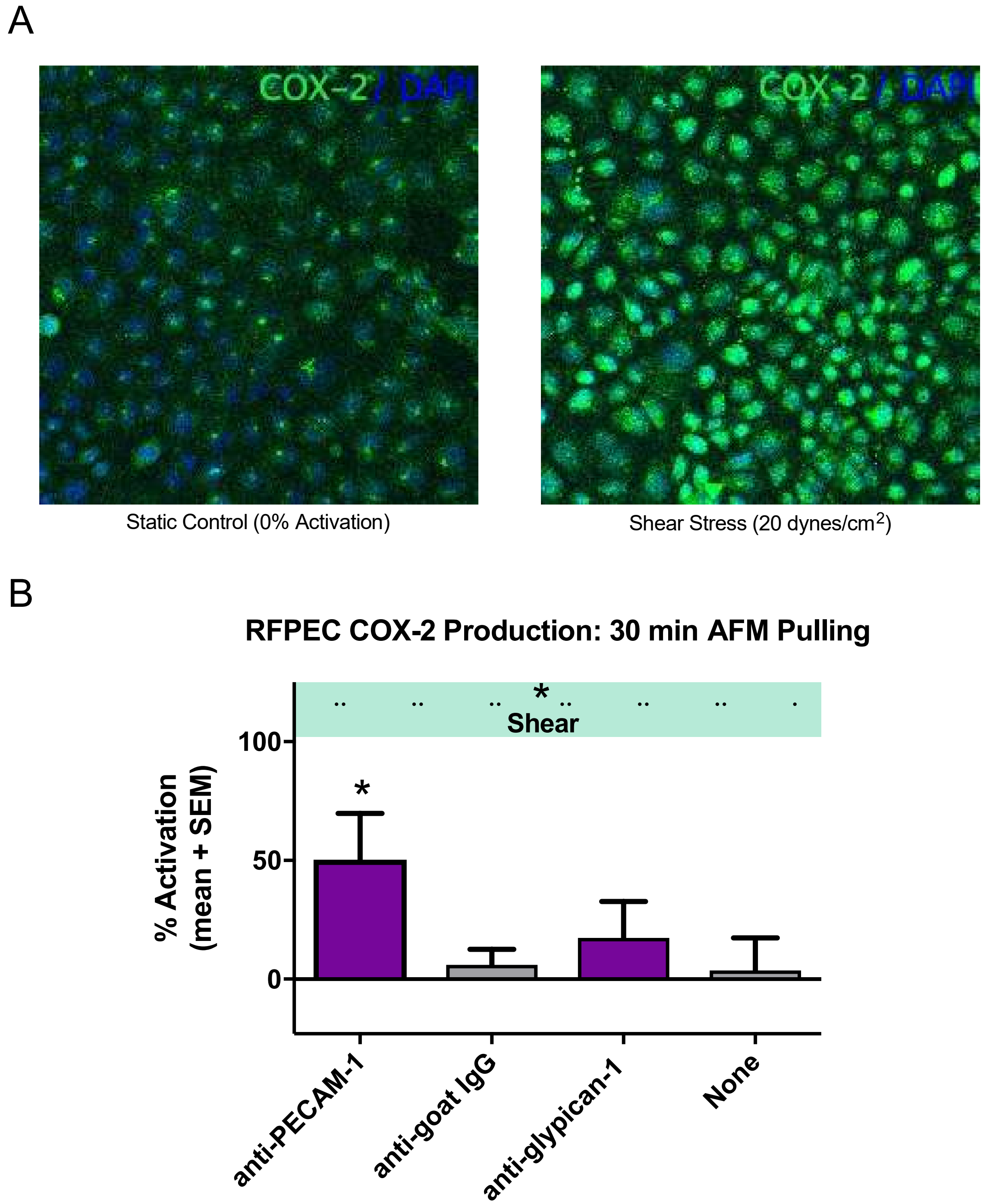

Figure S5. AFM Pulling with PECAM-1 Antibody Induces Increased COX-2 Production in RFPECs. A. Max intensity z-projections of static (left) and shear (right) RFPEC monolayers, with COX-2 (green) and DAPI (blue) fluorescent staining. **B.** RFPEC monolayers were probed with tipless cantilevers functionalized with goat PECAM-1 antibody (n = 8), normal goat IgG (n = 7), rabbit glypican-1 antibody (n = 8), or bare tipless cantilevers ('None' group, n = 7). Mean % activation and SEM are shown for COX-2 production in the bar graph. *P<0.05 versus static conditions (0% activation) using two-tailed one-sample t-test.

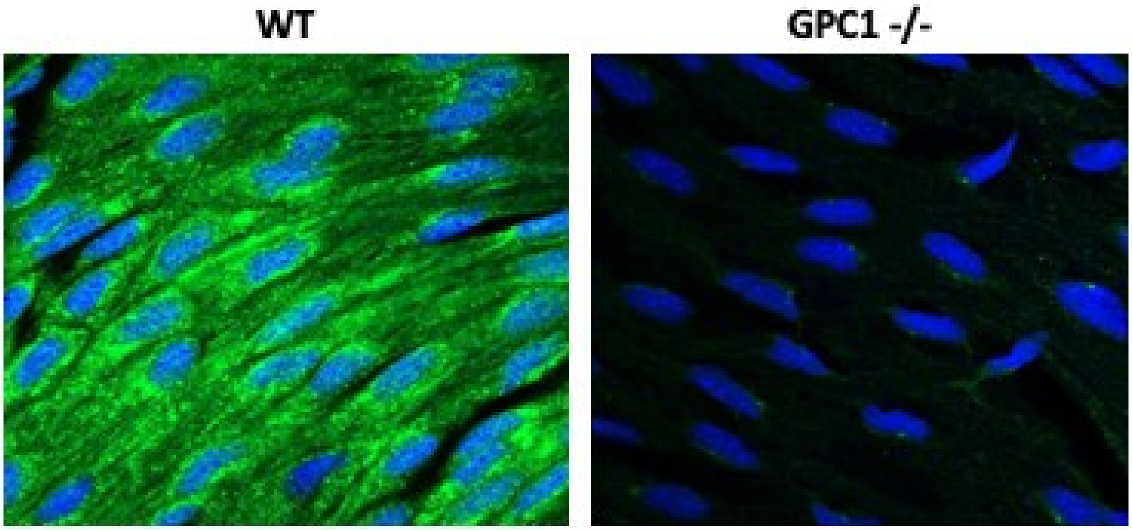

Figure S6. Gpc1-/- Mice Lack Glypican-1 Expression in Descending Aorta. WT and Gpc1-/- mice with glypican-1 (green) and DAPI (blue) fluorescent staining.

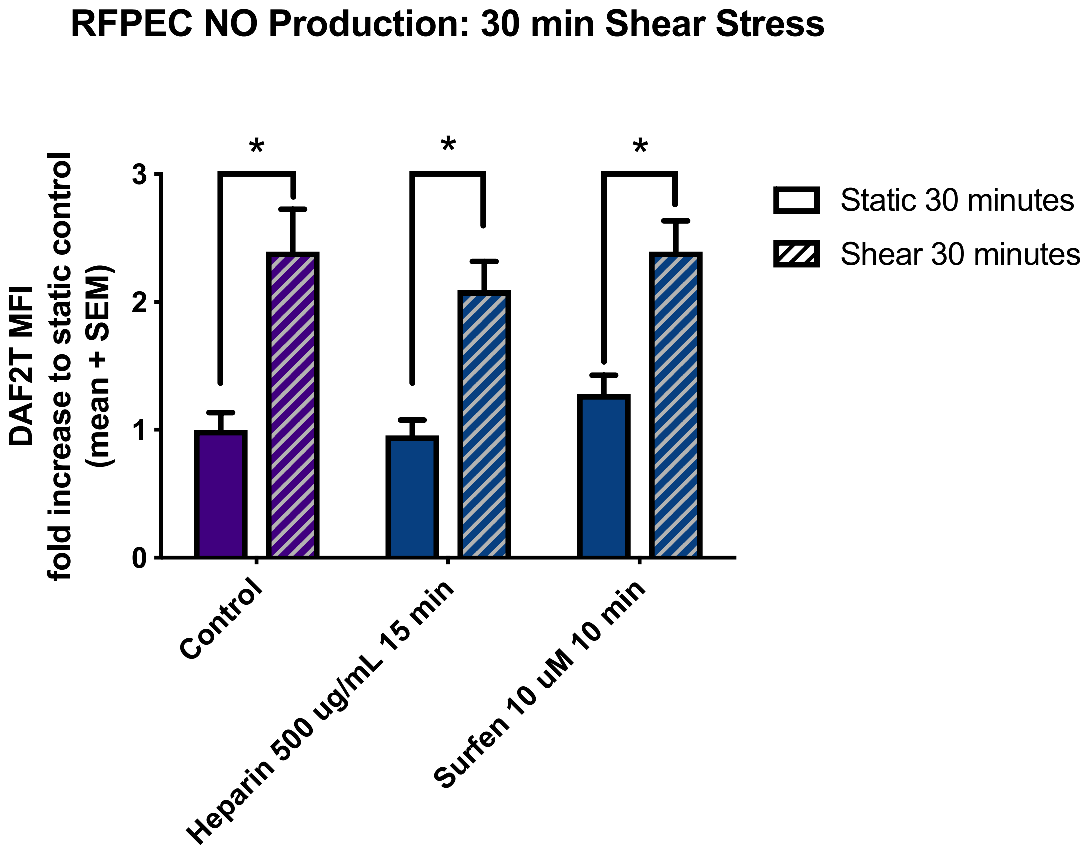

Figure S7. Heparin and Surfen Treatment Does Not Affect Shear-Induced NO Production at 30 minutes in RFPEC. Heparin (500 µg/mL) and surfen (10 µM) were used to treat RFPEC 10 minutes prior to fluid shear stress exposure to block shear-induced HS binding to PECAM-1. DAF-2 T levels were normalized to static control levels within each set. From left to right, n = 10, 16, 5, 10, 4, 7. *P<0.05 using two-way ANOVA followed by Sidak's multiple comparison's test.

Figure S8. Original blots with multiple exposures. Membranes were cut for blotting.

For manuscript FIGURE 3C:
BLOT 1: (top) anti-PECAM-1 (bottom) anti-beta-actin

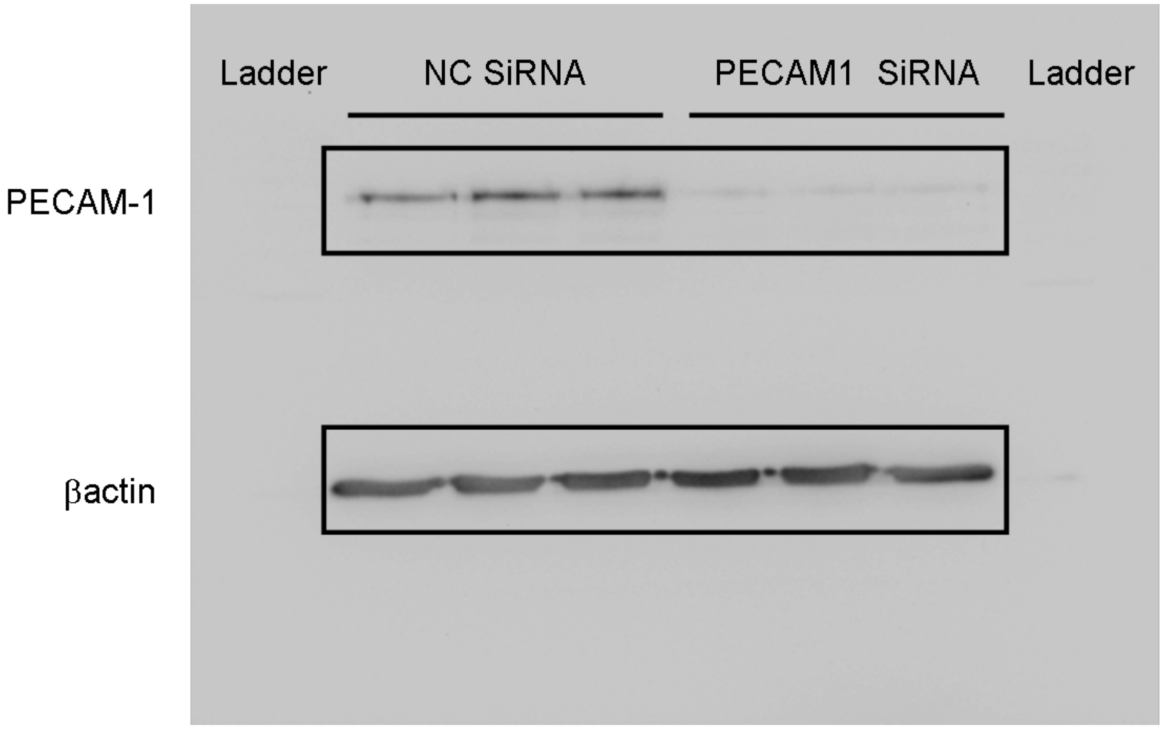

For manuscript FIGURE 6A:
BLOT 1 top: anti-phosphotyrosine from PECAM immunoprecipitation (cut membrane for blotting)
(exposure 1)

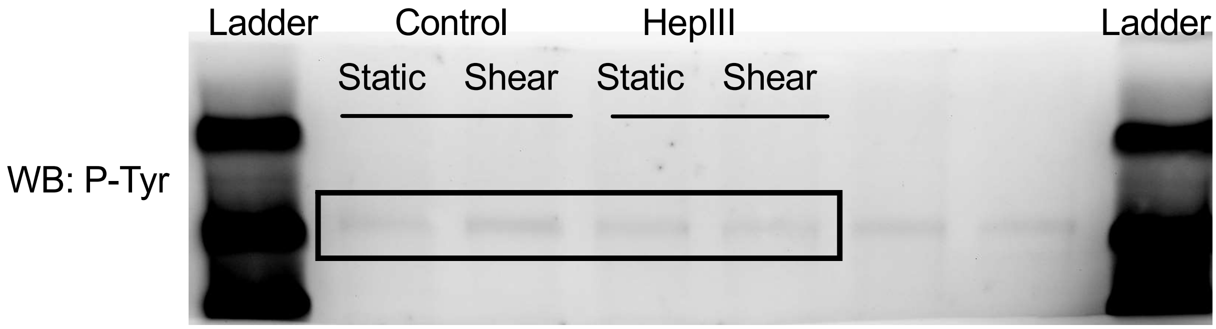

(exposure 2)

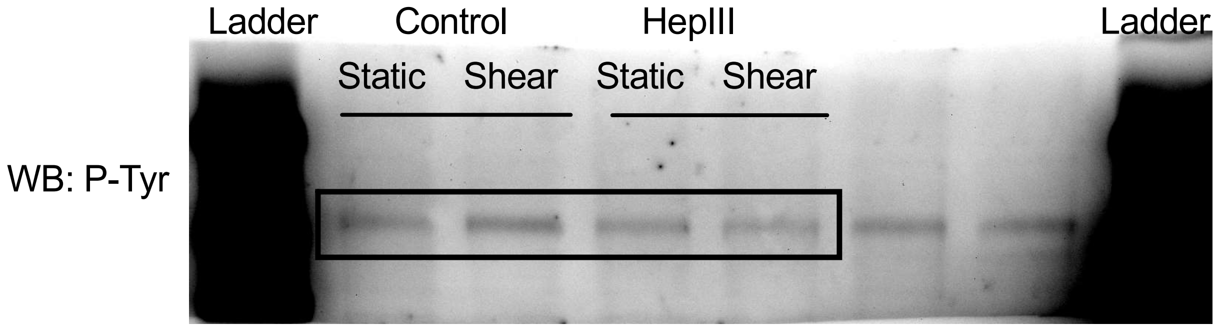

BLOT 1 bottom: anti-PECAM from PECAM immunoprecipitation (cut membrane for blotting)
(exposure 1)

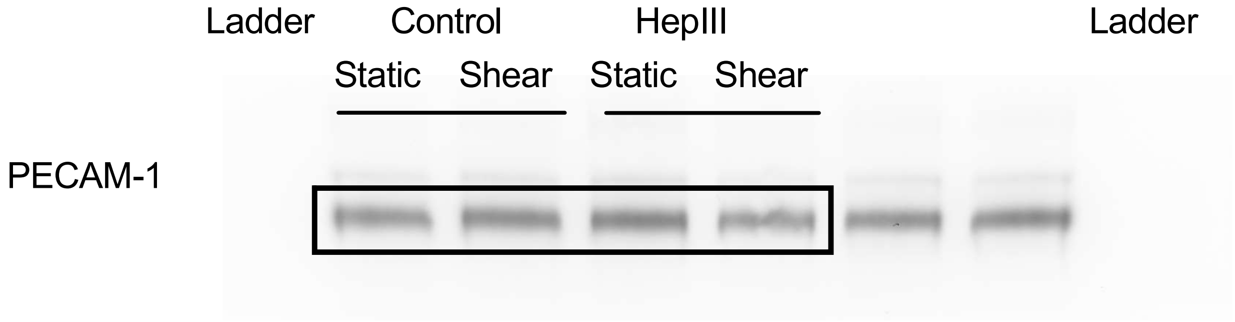

For manuscript FIGURE 6B:
BLOT 2 top: anti-phosphoTyr686 (cut membrane for blotting)
(exposure 1)

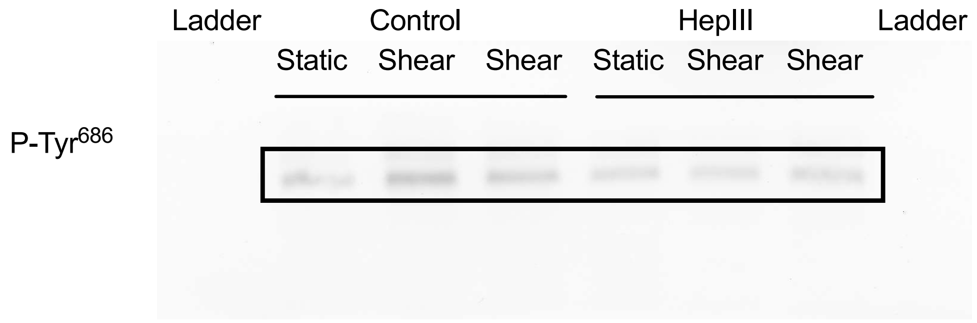

(exposure 2)

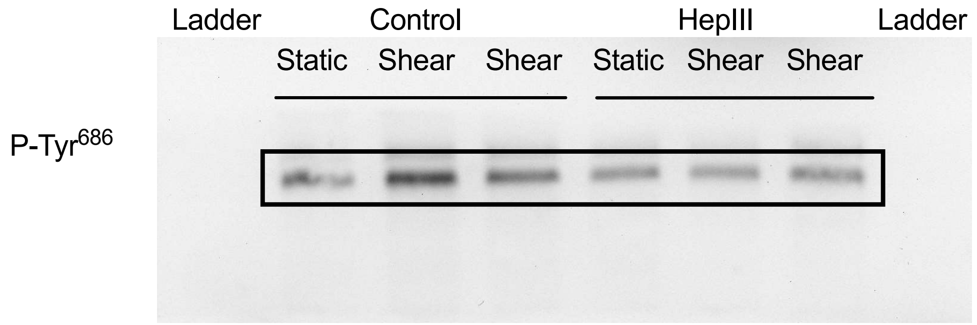

BLOT 2 bottom: anti-PECAM (cut membrane for blotting)
(exposure 1)

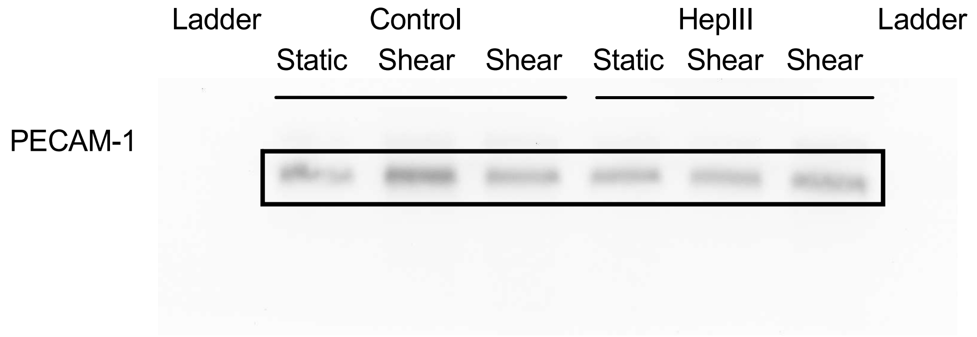

(exposure 2)

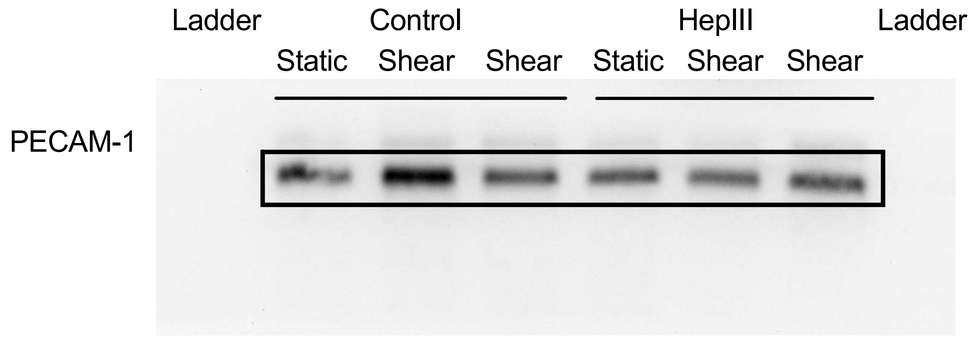

Supplement: Supplementary file 1 — Supplementary Information. [file 41598_2021_90941_MOESM1_ESM.pdf]
